# Consensus-led recommendations defining practical principles of achieving optimal surgical outcomes in robotic colorectal surgery in the Asia–Pacific region

**DOI:** 10.1007/s11701-022-01439-0

**Published:** 2022-06-30

**Authors:** A. C. Lynch, J. Ngu, S. S. M. Ng, S. Tsukamoto, A. Shiomi, X. Lai, J. Y. Wang, T. Scoble

**Affiliations:** 1 Sydney Adventist Hospital, Sydney, Australia; 2 Australian National University, Canberra, Australia; 3 Changi General Hospital, Singapore, Singapore; 4 The Chinese University of Hong Kong, Hong Kong, China; 5 National Cancer Centre, Tokyo, Japan; 6 Shizuoka Cancer Centre, Shizuoka, Japan; 7 Peking Union Medical College Hospital, Beijing, China; 8 Kaohsiung Medical University Hospital, Kaohsiung Medical University, Kaohsiung, Taiwan; 9 Ministry of Health and Welfare Pingtung Hospital, Pingtung, Taiwan; 10 Triducive Partners Ltd., St. Albans, UK

**Keywords:** Colorectal surgery, Robotic surgical procedures, Consensus, Integrated table motion, da Vinci Xi, Minimally invasive surgery

## Abstract

Recent innovations within the field of robotic surgery have particular relevance to colorectal surgery. Although a robotic approach has been associated with satisfactory outcomes, there remains a wide variation in levels of adoption. In particular, this study focuses on patient positioning, docking, and table placement, with the intent of understanding the strength of opinion of colorectal surgeons in the Asia–Pacific region to the practical application of these developments to achieve optimal surgical outcomes. Using a modified Delphi methodology, a steering group of colorectal surgeons with experience in robotic surgery from across the Asia–Pacific region identified 35 consensus statements. An online 4-point *Likert* scale questionnaire was distributed to surgeons in the Asia–Pacific region using convenience sampling. Respondents were excluded from further analysis if they did not perform colorectal surgery or had no experience in robotic surgery. A total of 140 responses (71.8% response rate) were received between August and October 2021. 22 statements attained a very high degree of agreement (≥ 90%). High agreement (< 90% and ≥ 75%) was achieved in another 12, and one failed to meet the consensus threshold (< 75%). A set of five recommendations were developed based on these results. The high levels of agreement demonstrate recognition amongst colorectal surgeons within the Asia–Pacific region of the potential advantage of recent improvements in robotic surgery technology to further improve surgical outcomes. The recommendations may inform a set of practical principles to help standardise the use of colorectal robotic surgery, which may also be relevant to other surgical fields.

## Introduction

Various technical challenges to conventional laparoscopic colorectal surgery have been reported. These include a high body mass index (BMI), narrow pelvis, bulky tumours, and low rectal tumours [1, 2]. Being able to alleviate these challenges has been one of the main benefits of robotic surgery (RAS). RAS provides surgeons with a greater range of precise and complex motions through instrument articulation and tremor filtration. Together with an immersive high-definition 3D view of the surgical field and improved ease-of-access, modern RAS systems facilitate increasingly complex minimally invasive procedures [2]. Since Weber et al. published their experience with the first two robotic colectomies in 2002 [3], numerous studies have demonstrated a significant reduction in the rate of conversion to open surgery, even in high-risk patients [4, 5]. Reductions in length of stay (LOS), number of postoperative days to first oral intake, and time to recovery of bowel function have also been described [1, 4, 6, 7].

Despite these advantages, the adoption of RAS for colorectal surgery was initially limited. One of the reasons is the multi-quadrant nature of colorectal procedures. RAS surgeons often have to improvise their port placement and docking strategies to complete multi-quadrant procedures. This led to variations in practice, differing outcomes, and suboptimal propagation of robotic techniques [5]. The need for complex hybrid laparoscopic-robotic approaches or intraoperative redocking also contributes to the operative time and learning curve of colorectal RAS [4].

The introduction of the da Vinci Xi system (Intuitive Surgical Inc., Sunnyvale, California, USA) in 2014 was intended to address this issue [8]. The Hillrom TS7000dV surgical table (Hill-Rom Holdings Inc., Chicago, USA) was subsequently developed to complement the da Vinci Xi. With Integrated Table Motion (ITM) technology, the TS7000dV can connect wirelessly to the da Vinci Xi allowing intraoperative adjustments to table positioning eliminating redocking of the robotic patient cart. This overcomes a major limitation of older systems where it was not possible to change the position of the operating table or patient once the patient cart was docked, potentially leading to compromises in patient positioning during surgeries that spanned multiple quadrants [8–10]. In addition, it is recognised that the steep Trendelenburg position that patients may be placed in can be associated with adverse effects such as oedema of the upper airway, reduced pulmonary compliance, elevated intraocular pressure, soft-tissue injuries, and haemodynamic and neurological complications [9, 11]. By enabling the patients to be repositioned easily throughout surgery, ITM potentially limits the amount of time that a patient is exposed to such risks.

However, the TS7000dV is not routinely acquired with the da Vinci Xi, in part because it is manufactured by a different company. The hospital administration and stakeholders that decide on equipment purchase may not be cognisant of the synergistic role that the TS7000dV has.

The intent of this study was to explore the opinions of colorectal surgeons in the Asia–Pacific region regarding the key challenges in implementing RAS in colorectal surgery, principles for best-practice, and the potential for patient positioning, docking and ITM to contribute to improvements in surgical outcomes. From this, the steering group aimed to formulate recommendations to improve patient care through the standardisation of procedures using RAS and ITM.

## Method

A steering group was formed of seven colorectal surgeons with experience in robotic colorectal surgery (CR RAS) surgery (with and without ITM experience) from across the Asia–Pacific region. The group met virtually in June 2021 to discuss the challenges surrounding the delivery of robotic colorectal surgery, how patient and procedural selection decisions for CR RAS are made, and what opportunities exist to optimise outcomes. From this discussion, the group agreed on four key themes:

1. Challenges in CR RAS
2. Good practice principles in CR RAS
3. Surgical table integration with robotic system
4. Training & communication opportunities.

Employing a modified Delphi method, these themes were discussed by the group and 35 consensus statements were developed for testing across a wider surgical audience (Table 1) which were used to form an online survey. The steering group identified 195 eligible respondents across the region. The survey was distributed on this basis via a convenience sampling method [12] to surgeons with and without experience in ITM. Responses from the survey were screened and respondents who did not perform colorectal surgery or had no experience in RAS were excluded from analysis. The threshold for consensus agreement was set at 75% or greater. Consensus would then be defined as ‘high’ at < 90% and ≥ 75% and ‘very high’ ≥ 90%.

**Table 1:** Defined consensus statements and corresponding levels of agreement

No.	Statement	Score (%)
Topic 1: Challenges in colorectal (CR) robotic surgery (RAS)		
1	Multi-quadrant minimally invasive surgery (MIS) requires intraoperative changes in table positioning	91
2	Colorectal (CR) procedures involve a broad operative field and several anatomical targets that span different abdominal quadrants	95
3	Patient/table positioning is often needed to visualise target anatomy and optimise access during colorectal robotic surgery (CR RAS)	76
4	Previous generations of robotic surgical systems required either a hybrid laparoscopic-robotic approach or intraoperative redocking to complete multi-quadrant surgeries	92
5	Surgeons find undocking and redocking to improve target organ access/visibility difficult and time-consuming during CR RAS	81
6	The optimal position of the patient changes as the procedure progresses and outcome may be compromised if table repositioning is avoided	84
7	A robotic system without integrated table motion (ITM) requires significant operating room (OR) staff time and input to achieve optimal patient positioning during CR RAS	79
8	Retracting bowel against gravity to improve access during CRS increase the risk of damage to the bowel	79
9	Optimal patient positioning in MIS colorectal surgery can involve extremes of table tilt/trend	98
10	A robotic system (without ITM) requires frequent undocking/redocking to manage extremes of positioning in higher risk patients or in longer cases	66
Topic 2: Good practice principles in colorectal (CR) robotic surgery (RAS)		
11	It is ideal to maintain visibility and access to the target organ throughout CR RAS surgery	99
12	Patient/table repositioning to maintain visibility and access to target anatomy during CR RAS should be done as frequently as is required	91
13	Facilitating a seamless table position change during CR RAS can maintain view and optimal access	98
14	Facilitating a seamless table position change during CR RAS can avoid disruption to the flow of surgery and distraction to the surgeon	96
15	Facilitating a seamless table position change during CR RAS can reduce the overall procedure time	99
16	Certain patient cohorts who are unable to tolerate prolonged periods of extreme positioning would benefit from intraoperative repositioning	96
Topic 3: Surgical table integration with robotic system		
17	ITM reduces the need to undock and redock the patient cart to reposition the table during CR RAS	96
18	ITM reduces the time associated with table/patient repositioning	98
19	The surgeon is more aware of the intraabdominal view during table/patient repositioning when using ITM	91
20	ITM helps to overcome the limitation of the fixed position of the patient on the table by offering flexibility to dynamically improve the operative field of view during CR RAS	96
21	ITM helps reduce the risk of unintended bowel injury by reducing the requirement to retract bowel against gravity	85
22	Splenic or hepatic flexure mobilisation is easier with ITM	87
23	ITM has a significant role to play in optimising multi-quadrant colorectal surgery	97
24	The appropriate use of ITM in CR RAS can improve surgical outcomes	84
25	The appropriate use of ITM in CR RAS can improve surgical efficiency	97
26	The appropriate use of ITM in CR RAS can reduce overall RAS procedure times	97
27	Patient outcomes can be improved by appropriate use of ITM in CR RAS to minimise the amount of time spent in extreme positions of tilt	92
28	ITM helps surgeons standardise procedures by providing a precise real-time display of the tilt/trend status	91
29	ITM can provide the surgical team with greater confidence to perform longer/more complex procedures	86
Topic 4: Training and communication opportunities		
30	Robotics facilitates the adoption and applicability of MIS CR surgery by making components of the procedure easier	90
31	ITM can help shorten the learning curve for splenic/hepatic flexure mobilisation by facilitating intraoperative changes in patient positioning	89
32	ITM provides continuous real-time readout of table positioning status which can help enhance teaching and video review	89
33	ITM improves communication between the surgical and anaesthetic teams by providing a precise real-time display of the tilt/trend status	87
34	The environment where ITM is used should be safe and all OR staff should be trained to achieve competency	99
35	ITM increases the utility of current robotic systems	92

Respondents were offered a four-point *Likert* scale to rate their agreement with each statement, ranging across ‘strongly disagree’, ‘tend to disagree’, ‘tend to agree’, and ‘strongly agree’. The online survey collected some demographic data on respondents including: country of work, if they performed colorectal surgery, the type of colorectal surgery performed, the number of robotic surgeries performed each month, and their years of experience in robotic colorectal surgery. Respondents were also asked if they currently perform minimally invasive surgery (MIS) RAS with Intuitive da Vinci surgical systems, the model of da Vinci system used, and if they had experience in using ITM. Completed surveys were collated anonymously before being analysed by an independent facilitator (Triducive Partners Limited, St Albans, UK) to produce an arithmetic agreement score for each statement.

No patients were involved in this study and Institutional Review Board approval was not necessary.

## Results

**Fig. 1:**
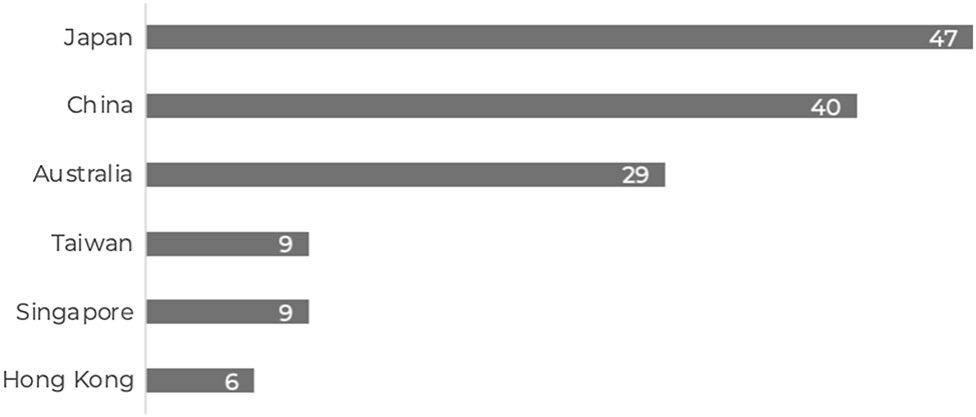
Total number of qualifying respondents per country after exclusion criteria applied

A total of 140 responses (71.8% response rate) that met inclusion criteria were collected between August and October 2021 from across six countries in the Asia–Pacific region (Fig. 1). All qualifying respondents currently perform colorectal surgery using MIS RAS with Intuitive da Vinci surgical systems. Respondents’ country is outlined in Fig. 1. Fifty seven (41%) had ITM experience with the da Vinci Xi system. Qualifying surgeons have been performing MIS RAS for a mean of 3.4 years (range 0–22 years), performing a mean of 3.9 procedures per month (range 1–65). Given the size of the sample, only overall results are reported.

The responses were generally consistent across the majority of statements; 22 attained very high agreement and 12 attained high agreement. Only one statement failed to reach the threshold for consensus. Given the high response rate and significant levels of agreement, the steering group agreed that further rounds of survey were not required.

**Fig. 2:**
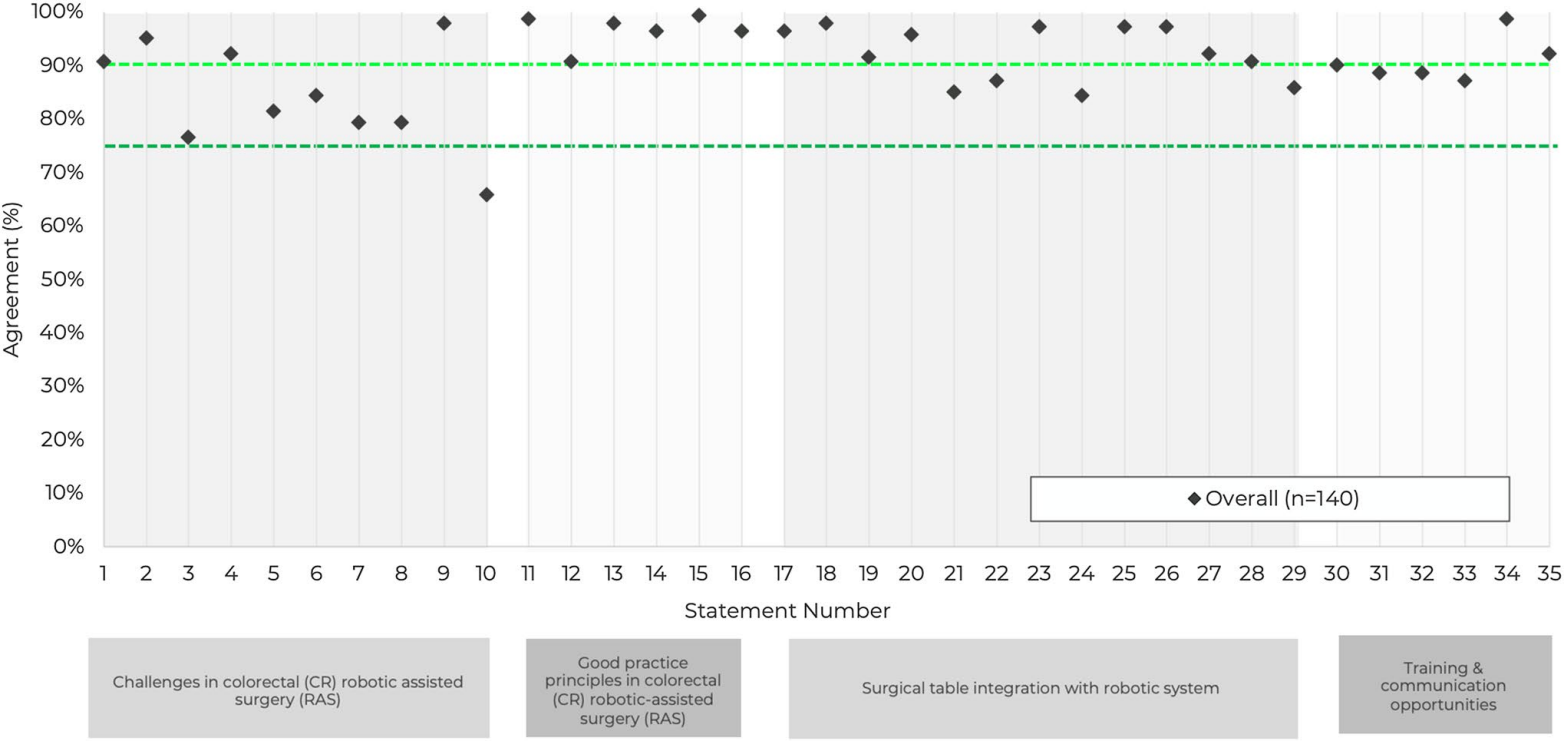
Combined consensus scores by statement. Note: the dark green horizontal line represents consensus agreement threshold of 75%, and the light green line indicates the threshold for very high consensus (90%)

The level of agreement for each statement is shown in Table 1, with a graphical representation of the overall results shown in Fig. 2.

**Fig. 3:**
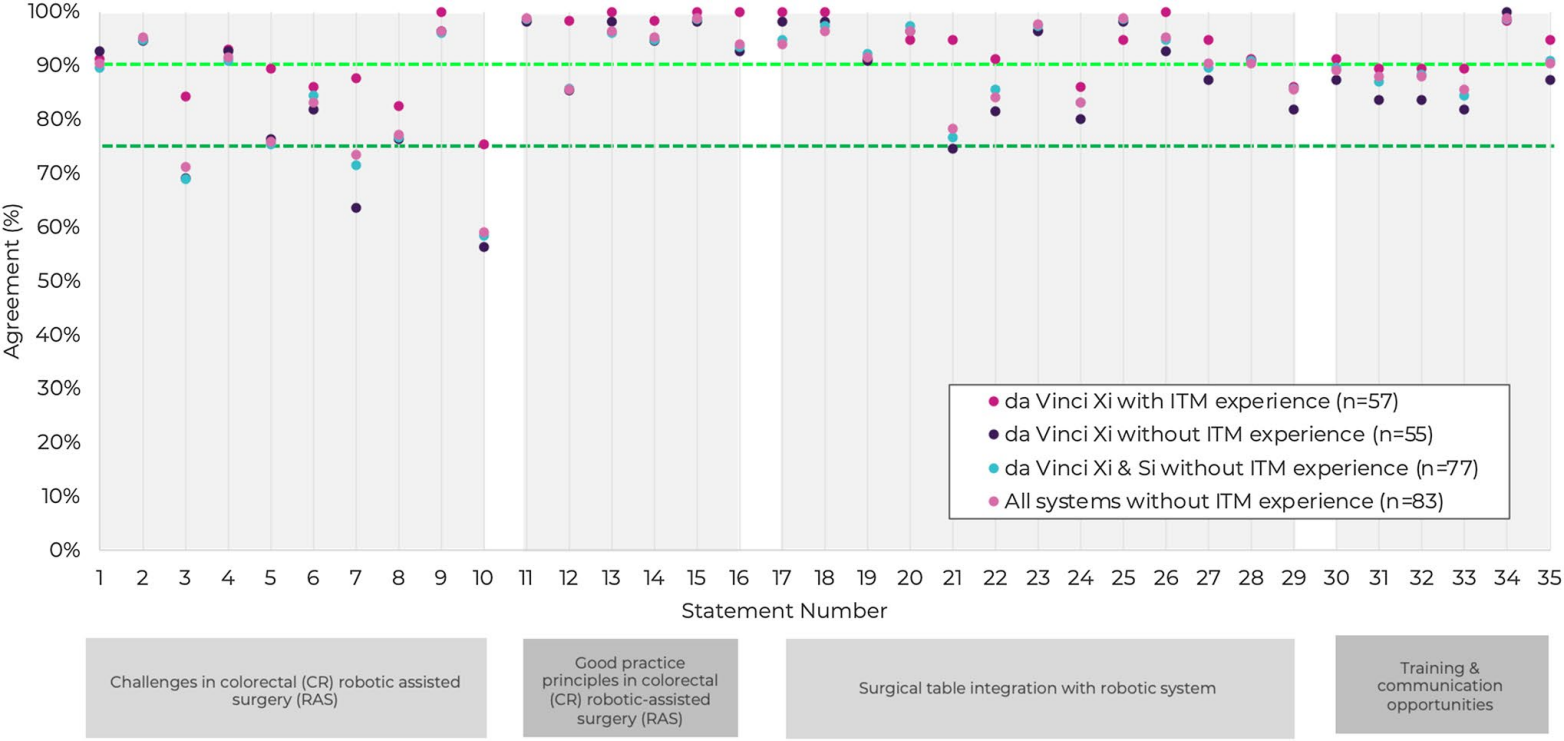
Consensus scores displayed by experience with robotic system and ITM experience. Note: The dark green horizontal line represents consensus agreement threshold of 75%, and the light green line indicates the threshold for very high consensus (90%)

Subgroup analysis of the data was performed by stratifying the respondents according to the robotic system they were experienced in (Si, Xi, or both) and whether ITM was incorporated to their practice (Fig. 3). This demonstrates a concordance of opinion across groups, except for most notably Statement 10 where surgeons without ITM experience did not feel that frequent undocking/redocking manoeuvres to manage extremes of positioning were required when using robotic systems without ITM in higher risk patients or in longer cases.

## Discussion

### Challenges in colorectal (CR) robotic surgery (RAS)

In contrast to prostatectomy and hysterectomy, which occur solely in the pelvis, a common feature in CR surgery is a broad operative field [4, 8]. Respondents strongly agree that multi-quadrant surgery presents an additional challenge to surgeons by requiring changes in table positioning and redocking of the robot (Statements 1, 2, 3, 5, and 6). This is particularly relevant to older model robotic systems, and systems that lack ITM capability (Statements 4 and 7). Changes in table tilt may also be necessary to relieve the physiological impact of extreme positioning and to make use of gravity for the displacement of abdominal viscera (Statements 8 and 9).

Statement 10 (63%) failed to reach the overall threshold for consensus. The steering group hypothesise that this may be due to the inclusion of the word ‘frequent’. Omission of the word or a clearer quantification may have led to higher agreement. In addition, the subgroup analysis in Fig. 3 shows a clear divergence in opinion between surgeons with access to ITM and those without. It is probable that those surgeons without access to ITM may modify their practices, such as utilising a hybrid approach to avoid redocking, and therefore disagree with statement 10. Conversely, surgeons operating with ITM were at liberty to make adjustments to table tilt as often as necessary, therefore appreciating the need for frequent redocking in the absence of ITM.

### Good practice principles in colorectal (CR) robotic surgery (RAS)

The high agreement levels for Statements 11–16 indicate that these principles are accepted by most surgeons. In particular, the responses to statement 16 indicate that surgeons are aware of the benefits of intraoperative repositioning in high-risk patients. However, coupled with the responses to statements 10, 14, and 15, it would appear that surgeons operating without ITM do compromise on ideal patient positioning due to the disruptions in surgery that result from redocking.

### Surgical table integration with robotic system

The consensus demonstrated by respondents for statements 17–29 demonstrates the precognition of potential benefits of ITM. Agreement with statements 23 and 24 suggests that surgeons, regardless of experience with ITM, acknowledge that outcomes can be improved by incorporating the technology. There is also clear agreement that the use of ITM improves surgical efficiency and procedure times (statements 25 and 26, both 97%).

The results for statement 23 (97%) are congruous with the existing literature that suggests ITM improves surgical efficiency, increasing the utility of RAS in multi-quadrant surgery [8–10, 13].

### Training and communication opportunities

Respondents recognise the benefits of ITM in facilitating communication within the operating room (OR), and in training of the robotic surgeon.

### Recommendations

On the basis of the results achieved across all 35 statements, the authors offer the following recommendations:

1. Patient/table repositioning to maintain visibility and access to target anatomy should be performed as frequently as possible. The same should be applied to patients who are unable to tolerate prolonged periods of extreme positioning during colorectal RAS.
2. Seamless changes in table position during colorectal RAS would be ideal to reduce procedure time.
3. If available, ITM should be used to optimise OR efficiency, surgical team communication, and ultimately patient outcomes.
4. ITM should be considered essential in maximising the utility of the da Vinci Xi system for multi-quadrant procedures.
5. Advice from surgical teams should be sought when making decisions regarding the requirement and utility of robotic systems and their ancillary equipment.

### Strengths and limitations

The design of this study benefitted from an ensemble of steering group members from across the Asia–Pacific region. Seeking responses from these countries also serves to address any potential country/market-specific bias. The large sample collected over the course of the study provides a representative view of the opinions held by surgeons within the field of CR RAS. The strict inclusion criteria ensured that only experts in the field were consulted.

While the amount of experience each surgeon had with ITM may have influenced their response to certain questions, this was not apparent in the subgroup analyses. Using a convenience sampling method to collect responses, it is possible that the results are subject to motivation bias. This was mitigated by obtaining responses over a 3 month period and using a set of questions that had been validated by the group of expert colorectal surgeons that formed the steering group. Given the selection method, bias may have been introduced to the findings. Therefore, repeating the study with a set of respondents picked by an independent third party would be beneficial to determine the strength of opinion.
